# Serotype IV *Streptococcus agalactiae* ST-452 has arisen from large genomic recombination events between CC23 and the hypervirulent CC17 lineages

**DOI:** 10.1038/srep29799

**Published:** 2016-07-14

**Authors:** Edmondo Campisi, C. Daniela Rinaudo, Claudio Donati, Mara Barucco, Giulia Torricelli, Morven S. Edwards, Carol J. Baker, Imma Margarit, Roberto Rosini

**Affiliations:** 1 GSK Vaccines s.r.l., Siena, Italy; 2 Sapienza, Università di Roma, Rome, Italy; 3 Department of Computational Biology, Research and Innovation Centre, Fondazione Edmund Mach, San Michele all’Adige, Italy; 4 Department of physics “Enrico Fermi”, University of Pisa, Pisa, Italy; 5 Department of Pediatrics, Baylor College of Medicine, Houston, Texas, USA; 6 Department Molecular Virology and Microbiology, Baylor College of Medicine, Houston, Texas, USA

## Abstract

*Streptococcus agalactiae* (Group B *Streptococcus*, GBS) causes life-threatening infections in newborns and adults with chronic medical conditions. Serotype IV strains are emerging both among carriers and as cause of invasive disease and recent studies revealed two main Sequence Types (STs), ST-452 and ST-459 assigned to Clonal Complexes CC23 and CC1, respectively. Whole genome sequencing of 70 type IV GBS and subsequent phylogenetic analysis elucidated the localization of type IV isolates in a SNP-based phylogenetic tree and suggested that ST-452 could have originated through genetic recombination. SNPs density analysis of the core genome confirmed that the founder strain of this lineage originated from a single large horizontal gene transfer event between CC23 and the hypervirulent CC17. Indeed, ST-452 genomes are composed by two parts that are nearly identical to corresponding regions in ST-24 (CC23) and ST-291 (CC17). Chromosome mapping of the major GBS virulence factors showed that ST-452 strains have an intermediate yet unique profile among CC23 and CC17 strains. We described unreported large recombination events, involving the *cps* IV operon and resulting in the expansion of serotype IV to CC23. This work sheds further light on the evolution of GBS providing new insights on the recent emergence of serotype IV.

The human pathogen *Streptococcus agalactiae* (also referred to as Group B *Streptococcus* or GBS) is a common cause of life-threatening bacterial infections in neonates and infants^1^. It can also cause disease in adults, particularly among older adults (≥65 years) and those with underlying medical conditions such as diabetes, cancer, cirrhosis and HIV infection^2^. The incidence of adult disease has markedly increased and now, in the Unites States, it contributes nearly 90% to the total burden of GBS disease^3^. Common clinical syndromes of GBS infection include sepsis and meningitis in infants and bacteremia, skin and/or soft-tissue infections and pneumonia in adults. GBS is also a commensal organism and rectal and vaginal colonization, affecting about 30% of healthy women, is the main risk factor for early onset neonatal infection, occurring between days 0 and 6^1^.

The GBS capsular polysaccharide (CPS) represents a major virulence factor, interfering with phagocytic clearance. It induces protective immunity and is considered an excellent target for the production of vaccines when conjugated to carrier proteins^4^. Ten CPS types have been identified, and five of these (Ia, Ib, II, III and V) account for the majority of disease both in neonates and in adults, although with significant differences in their relative distribution^5^. Serotype III is the most prevalent among neonatal invasive strains. In particular, a hypervirulent type III lineage belonging to Clonal Complex 17 (CC17) is responsible for the majority of late onset disease (LOD) cases, occurring between 7 and 89 days of age and often associated with meningitis^6^,^7^. Similar serotype distribution data have been reported globally^5^,^8^, although changes in the epidemiology have occurred over the last 30 years, including the emergence of type V strains after 1985^9^. An increase in the formerly rare serotype IV among adult invasive disease strains was first appreciated in a US surveillance study between 1999–2005^2^, and confirmed in Norway^10^, Ireland^11^, and Canada^12^,^13^. Further, a potential increase in type IV isolates was reported among colonized women between 2004 and 2008 in Minnesota^14^, invasive neonatal infections during 2000–2010 in the same setting^15^ and LOD cases between 2006 and 2008 in Southern Brazil^16^.

A recent study described a putative capsular switch from CPS type III to IV within the hypervirulent CC17^17^. The resulting type IV Sequence Type (ST)-291 clone that is a single locus variant of ST-17, most probably evolved from a single recombination event in a ST-17 genetic background due to the exchange of a 35.5 kb DNA fragment containing the entire *cps* operon. The exchange of large chromosomal elements spanning hundreds of kilobases between unrelated strains through conjugation and homologous recombination has been shown as one of the major forces driving GBS evolution^18^. Interestingly, a recent Portuguese study described a remarkable increase (20-fold) in serotype IV frequency and registered that ST-291 strains accounted for 10% of the whole collection^19^. Type IV invasive strains belonging to ST-291 have been also isolated in Taiwan^20^, US^15^, Ireland^11^, and Canada^21^. Besides ST-291 (CC17), the main circulating STs among serotype IV isolates include ST-452 in CC23, ST-459 and ST-196 in CC1^21^.

We performed whole genome sequencing analysis of a diverse collection of type IV GBS isolates with the aim of investigating their phylogenetic relationship, with a particular focus on strains belonging to the ST-452 lineage.

## Results

### Whole genome sequencing and *in silico* characterization of type IV GBS isolates

To investigate the genetic organization of the GBS serotype IV population, 70 clinical isolates were subjected to whole genome sequencing (Supplementary Table S1). As serotype IV is uncommon among circulating GBS human isolates, we constructed our strain collection selecting type IV GBS from different epidemiological studies and geographical areas to obtain a large collection of serotype IV GBS. Twenty-eight strains were recovered from adult invasive disease, 6 from neonatal invasive disease and 36 from colonized healthy women. Thirteen additional type IV genomes publicly available from the NCBI database were included in the analysis (Supplementary Table S2). The 83 available genome sequences first underwent an *in silico* typing process to confirm the presence of a canonical serotype IV-specific *cps* operon, to assign an ST and the relative CC to each strain, and to study the distribution of the Pilus Islands (PI).

Among the 83 genomes of our dataset, 55 were CC1 diversified in 12 different STs, of which 18 belonged to ST-196, and 17 to ST-459. CC23 was represented by 23 strains, of which 20 were ST-452. Four strains belonged to the CC17 and were ST-291 or single locus variants (s.l.v). Finally, the collection included a single ST-10 strain (CC6-8-10). The allelic profile of the new identified STs is reported in Supplementary Table S3.

Among the pilus types, the combination of pilus island 1 plus 2a was found in the 56 strains belonging to CC1 or CC6-8-10, while pilus 1 plus 2b genotype was found in each of the 4 strains belonging to CC17. These two pilus combinations are indeed very common among GBS isolates^22^. Conversely, the PI-2b-only genotype, generally quite rare in the GBS population, was found in all 23 strains belonging to CC23 (Table 1), in agreement with what previously observed^21^.

### Phylogenetic analysis showed that ST-452 is a separate clade within CC23

**Figure 1:**
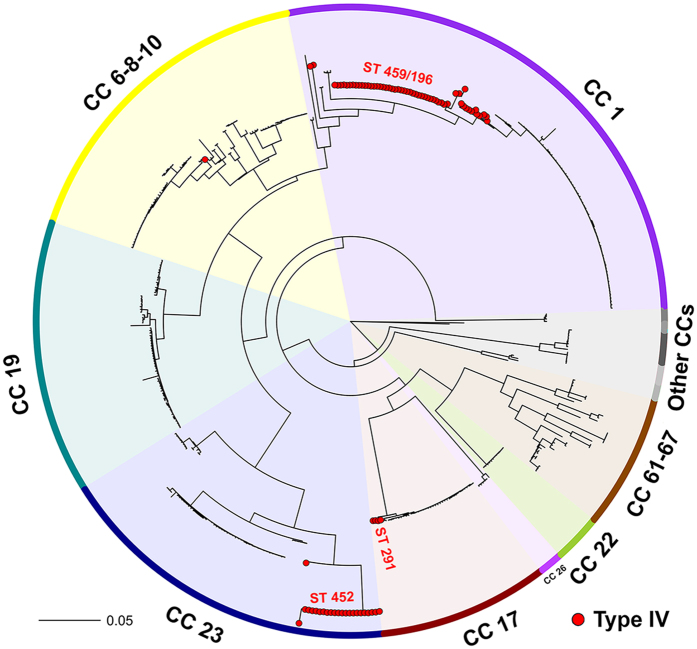
Phylogenetic distribution of type IV isolates within the GBS population. Phylogenetic tree based on the SNPs extracted from the core regions (24.7% of the reference genome, 2603V/R) of 491 GBS whole genome sequences, built using the FastTree algorithm. Colored ribbons and spikes indicate CCs assigned to the 7 major tree clades associated with GBS strains isolated from human or bovine hosts. Gray ribbons indicate clades corresponding to clonal complexes CC26, CC103, CC130, CC609, CC615, CC552 and CC261 mainly associated with strains isolated from frog, dog, dolphin and fish species. Red dots represent strains belonging to type IV capsular genotype.

To localize the major type IV circulating lineages in a complete GBS population tree, a phylogenetic analysis based on whole genome Single Nucleotide Polymorphisms (SNPs) was performed. For this purpose, the 70 newly sequenced type IV genomes were compared to 415 draft genome sequences gathered from the NCBI database and integrated by 6 genomes from our internal collection. A total of 16,480 SNPs were identified in the core genome (533,586 bp, 24.7% of the reference 2603 V/R genome) and used to construct a Maximum Likelihood phylogenetic tree (Fig. 1). Type IV isolates clustered in two major clades associated with CC1 (mainly ST-459 and ST-196) and CC23 (mainly ST-452) and one minor clade associated to CC17 (ST-291 and s.l.v.), in agreement with the MLST-based classification and with previous reports^14^,^15^,^21^. The three clonal complexes appeared to be distantly related in the global tree, consistently with former population-scale GBS phylogenies^23^,^24^.

It is worth noting that the single ST-10 strain of our type IV collection, clustering in CC6-8-10, shared no recent common ancestors with any other type IV.

### Horizontal exchange of a large genomic portion between ST-24 and ST-291 gave rise to ST-452

**Figure 2:**
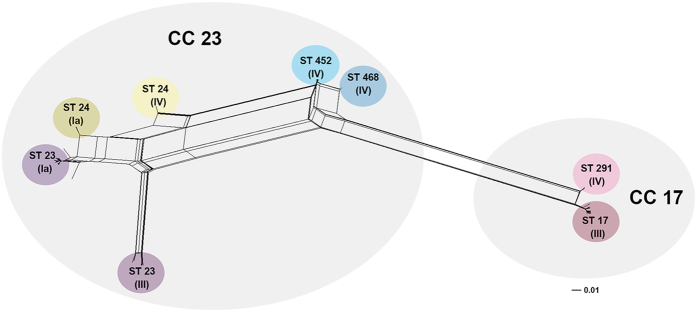
Phylogenetic network of CC17 and CC23 strains. Core genome based Neighbor-net split network depicting the impact of recombination on 130 GBS isolates belonging to CC17 and CC23. In this representation, all the conflicting phylogenetic signals due to each SNP are represented as alternative bipartitions that account for the non-tree-like structure of the inner part of the network. Strains from different STs are indicated and highlighted by colored circles. For each STs capsular genotype is indicated in brackets. Two grey shadings define the boundaries of the two CCs.

As reported above, the PI-2b-only was identified in all ST-452 genomes. Since this pathogenic island is typically associated with CC17 strains in combination with PI-1^22^, we hypothesized that the presence of this uncommon pilus variant in ST-452 could be the result of horizontal gene transfer (HGT) events between CC17 and CC23 strains. This assumption also could provide an explanation for the clear separation of ST-452 from the other lineages within CC23, as evidenced in the GBS population phylogenetic analysis (Fig. 1). Therefore, a more restricted analysis was conducted on a subset of 130 strains belonging to CC23 and CC17. A set of 14,790 SNPs was extracted from the resulting core genome (1,500,718 bp, 72.8% of the ST-452 reference genome, NGBS572) and was used to build a phylogenetic network by applying the neighbor net algorithm (Fig. 2). In this network, the nodes representing the ST-452 strains are connected both to the CC23 and CC17 strains, indicating that ST-452 has a tight phylogenetic relationship with both these CCs and suggesting a mosaic structure of this clone. Two other serotype IV strains belonging to CC23, and specifically ST-24 and ST-468, appeared to further link ST-452 to the rest of CC23 and to CC17, respectively. This highly connected area within the net further suggested that genomic recombination events could have occurred between strains from the two CCs.

**Figure 3:**
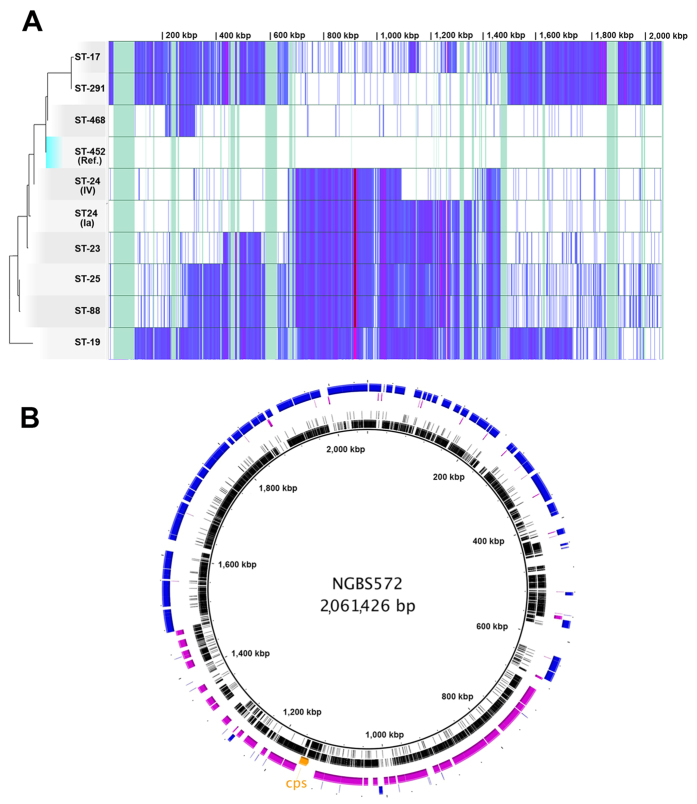
SNP density plot analysis of CC17 and CC23 strains and identification of recombination areas in serotype IV ST-452 NGBS572 strain. (**A**) Multiple-alignment view of the variant sites detected in the core genome of 9 GBS strains representative of CC17 and CC23 plotted against the complete chromosome of the ST-452 reference strain NGBS572. The reconstructed phylogenetic tree (rooted on a ST-19 strain) is paired with its corresponding rows in the multi-alignment based on SNPs identified on the 77.3% of the core genome. Variations with respect to the reference ST-452 strain (indicated as “Ref.” within the tree) are represented by a density plot that reveals the phylogenetic signatures of the resulting clades. Highly conserved stretches of sequence appear as blank regions that suggest common evolution, while a high density of SNPs (purple-blue regions) indicates distantly related regions. Green areas represent non-core regions excluded from the analysis. (**B**) Representative strains from CC17 (COH1, ST-17), CC23 (515, ST-23) were compared to ST-452 (NGBS572) genome for recombination detection. Foreign genomic segments identified in the NGBS572 (ST-452) genome by Bayesian analysis of recombination (BRATNextGen) coming from COH1 (ST-17) are represented in fuchsia while those from 515 (ST-23) are colored in blue. Polymorphisms identified in ST-17 strain COH1 (innermost ring) and ST-23 strain 515 (second ring) against the serotype IV ST-452 strain NGBS572 are depicted in black. Higher polymorphisms density at definite regions of the genome correlated with recombination areas identified among the strains. Genomic region encoding for the *cps* operon is colored in orange.

To seek definite evidence for putative horizontal gene transfer among genomes of the two CCs, we built a detailed SNPs density plot phylogram specific for CC17 and CC23. A new set of SNPs was extracted from the alignment of 10 genomes, including the genome sequence of the representative CC17 and CC23 strains (15,509 SNPs, core sequence 1,593,482 bp, 77.3% of the NGBS572 ST-452 reference genome). As evidenced in Fig. 3A, the reference ST-452 strain shared a high sequence identity in the central region of the chromosome (from base 670,507 to base 1,476,148) with CC17 strains (0.005 SNPs/100 bp for ST-291 and 0.069 SNPs/100 bp for ST-17) and with its s.l.v. ST-468 strain (0.003 SNPs/100 bp). By contrast, a 100-fold increase in SNPs density (0.430 SNPs/100 bp) respect to CC17 strains was observed in the rest of its genome sequence (up to roughly 60%, from base 1 to base 670,506 and from base 1,476,148 to base 2,061,426). In turn, this genome portion was almost identical to the corresponding region in CC23 ST-24 (type Ia) strain (0.006 SNPs/100 bp). These observations suggest the occurrence of a putative exchange of the large central region of the chromosome between a ST-24 (type Ia) and a ST-291 strain that likely triggered the rise of the ST-452 clone. In addition, ST-468 and ST-24 (type IV) presented a peculiarly intermediate SNP profile; the ST-468 strain had very similar SNPs density profile compared to the ST-452 reference strain, except for a region of 108,011 bp (0.412 SNPs/100 bp), which is almost identical to the corresponding region in ST-291 strains (from base 212,148 to base 320,159). The type IV ST-24 strain had a nearly identical SNP density profile to the other ST-24 strain (type Ia), except for a region (285,689 bp, from base 1,089,913 to base 1,375,602) that is highly similar to ST-452 (0.003 SNPs/100 bp). These evidences are suggestive of additional putative recombination events. Same results were observed by SNPs density analysis performed using a larger dataset of 130 CC17 and CC23 genomes (see Supplementary Fig. S1). Bayesian analysis performed on core genome alignments of reference CC17 and CC23 strains, identified recombination regions on ST-452 genome in agreement with those identified by SNPs density analysis. Moreover, this approach was able to identify further minor recombination events (Fig. 3B).

Finally, the recombination analysis was extended to the single ST-10 serotype IV strain of our collection, to investigate whether it could have acquired its *cps* operon by HGT. The Bayesian analysis identified four major regions in its chromosome that likely originated from ST-459 (see Supplementary Fig. S2). Particularly one of the identified putative recombination areas, spanning 85.4 kb harbors the *cps* IV operon.

### ST-452 strains have a unique combination of virulence factors

**Figure 4:**
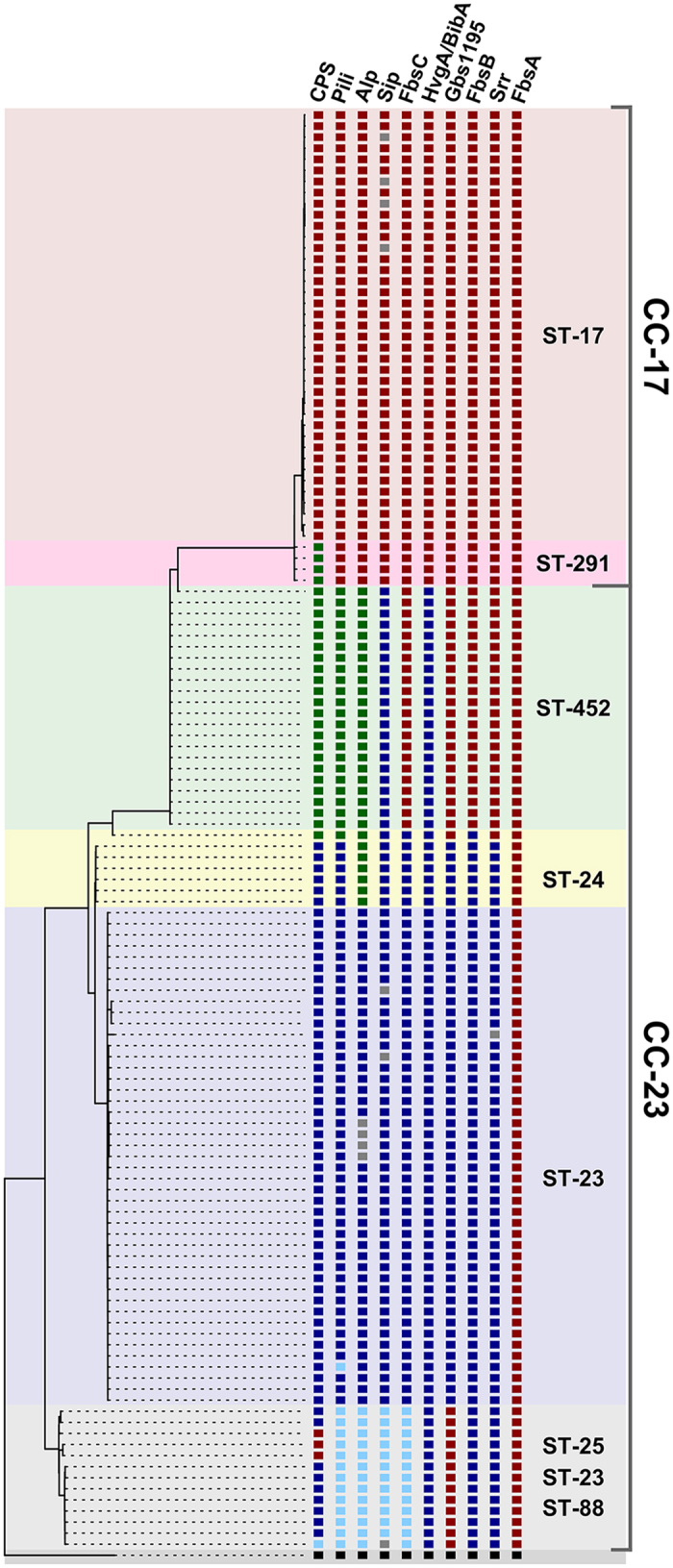
Distribution of allelic variants of major GBS virulence factors families. Capsule operon (CPS), Pilus island (Pili), Alpha proteins (Alp), Surface immunogenic protein (Sip), Gbs2018 (HvgA/BibA), Serine rich proteins (Srr), Fibrinogen-binding protein (FbsA, B and C), Gbs1195 among 130 isolates belonging to CC23 and CC17. STs (and their s.l.v.) within CC17 and CC23 are highlighted by colored areas. The dendrogram connecting the strains is the same used in Figure S1. For each virulence factor, allelic variants are represented in columns and differentiated by colored squares and defined as follows. **CPS**: red = III, green = IV, blue = Ia, cyan = II; **Pili**: red = 1 + 2b, green = 2b, blue = 2a, cyan = 1 + 2a; **Alp**: red = rib, green = c-alpha, blue = alp1 (epsilon), cyan = alp2; **Sip**: red = COH1, blue = 515, cyan = NEM316; **FbsC**: red = COH1, blue = 515, cyan = NEM316; **HvgA/BibA**: red = HvgA, blue = BibA; **Gbs1195**: red = Gbs1195, blue = Sag1127-2; **FbsB**: red = FbsB2b, blue = FbsB2; **Srr**: red = Srr2, blue = Srr1; **FbsA**: red = FbsA; **other**: grey = N.A., black = outgroup.

**Figure 5:**
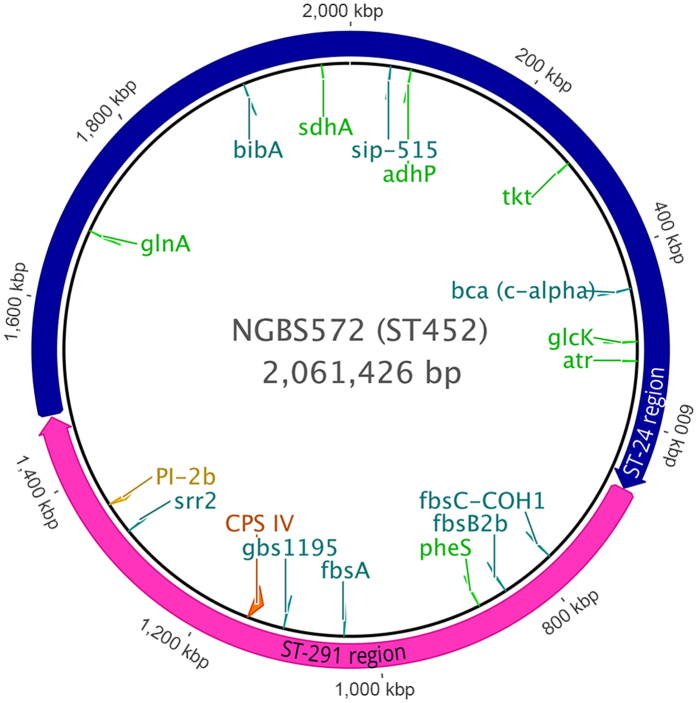
ST-452 chromosome map of major genetic features. Circular representation of the complete NGBS572 (ST-452) reference genome. MLST genes (green), *cps* IV locus (orange), PI-2b (yellow) and other virulence factors (turquoise) are represented. The putative ST-291 recombination region is marked in fuchsia while the ST-24 genome portion is colored in blue.

The genomes of all ST strains belonging to CC17 and CC23 underwent a second *in silico* typing process to evaluate the impact of the observed putative recombination events on the distribution of eight well described GBS virulence factors in addition to CPS and pili. These included the Alpha-like proteins (Alp)^25^, the Surface immunogenic protein (Sip)^26^, the surface adhesion protein HvgA/BibA^27^,^28^, the Serine rich (Srr) protein variants^29^, and fibrinogen-binding proteins like Gbs1195^30^,^31^, FbsA^32^, FbsB (also known as Fgag)^33^ or FbsC^34^. The result of this analysis was plotted on the phylogenetic tree built on the CC23-CC17 SNP density plot (Fig. 4). As expected, almost all strains belonging to a specific ST shared exactly the same combination of virulence factors. ST-291 and its s.l.v. only differed from their close relative ST-17 by having type IV instead of type III *cps* operon, otherwise ubiquitous in CC17. An exception to the above observed homogeneity is represented by strains within the deepest branch of CC23 and a single type IV ST-24 strain that showed a reassortment in their virulence factor profiles. The CC23 ST-452 lineage indeed presents a new and unique combination of virulence factors. More specifically it shares the Srr2 and the FbsB2b, FbsC-COH1, Gbs1195 fibrinogen binding protein variants with ST-17 and ST-291 and the BibA and Sip variants with ST-23/24. In addition, ST-452 has the ST-24 C-alpha protein variant. Finally, as reported above, the ST-452 is the only among the analyzed lineages carrying the PI-2b alone (Fig. 5).

## Discussion

*S. agalactiae* serotype IV is emerging as cause of invasive disease in adults, especially in Canada, accounting for 16.9% of invasive diseases during 2010–2014. In addition, several epidemiological studies have found significant proportions of this serotype among colonizing isolates from adults^14^,^16^,^35^,^36^. However, serotype IV is an uncommon cause of invasive disease in neonates^15^. Recent data reveal a relatively high level of genetic diversity among type IV isolates with the majority of strains belonging to two genetically divergent STs, ST-452 and ST-459 assigned to CC23 and CC1, respectively^21^. Of these, ST-459 was found to be the leading clone causing infections in non-pregnant adults in two different Canadian provinces^12^ and, based on genome analysis, it was proposed to derive from different recombination events with the closely related ST-1 within CC1.

We investigated the phylogeny and genomic variability of the GBS serotype IV population in an assorted collection composed of 70 newly sequenced strains isolated from infected adults, neonates and colonized pregnant women in Europe and US during the last 15 years, and 13 type IV strains for which the draft genomes were available in the NCBI database.

The analysis confirmed a high level of heterogeneity in this collection, with 19 STs grouped in 4 different CCs, of which ST-452 (CC23), ST-459 and ST-196 (CC1) predominated. Indeed, in agreement with previous reports^14^,^15^,^21^, serotype IV strains from different CCs appeared well separated, suggesting that the same capsular genotype is hosted in distinct genetic backgrounds.

Since the interest on the serotype IV has grown only recently, information before 2000 is relatively scarce. Based on MLST data reported in literature, it is interesting to note that the first described serotype IV strain belonged to ST-2, a core clade of CC1 that is represented by 7 strains in our collection^14^. Moreover, most type IV strains collected between 1988 and 2007 in Sweden^37^, France^38^ and Italy^39^ belonged to ST-196, thus confirming the prevalence of CC1 during this period^14^. The association of serotype IV isolates with other CCs was reported only later. The type IV ST-291 (CC17) was first reported in 2007^40^ and in the same year Héry-Arnaud and colleagues described a type IV strain belonging to ST-7 (CC6-8-10)^41^. Interestingly, the ST-291 has been shown to originate from a capsular switching event involving a ST-17 hypervirulent strain (CC17) and a ST-459 strain (CC1) that provided a 35.5 kb genomic segment comprising the type IV *cps* operon^17^.

We found that ST-452 could have originated from a relatively recent ancestor of the present ST-24 (CPS type Ia, CC23) strains that exchanged about 40% of its genome with an ST-291 (CPS type IV, CC17) strain by recombination. This event caused a further increase in serotype IV diversity by generating the ST-452 type IV lineage. This new clone gained a unique combination of virulence factors, partially sharing allelic variants with CC17 or CC23. Interestingly, none of the ST-452 strains of our collection had any known antibiotic resistance genes in their genomes, in contrast to those belonging to ST-459 (Supplementary Table S4) and in accord with a previous report^21^.

The analysis of the SNP density allowed us to identify two further large horizontal transfers that could have promoted the spread of serotype IV throughout the GBS population. In fact, the same mechanism likely acted on a ST-24 that exchanged a 285,689 bp chromosome tract, including the capsular operon, with an ST-452 strain. The second recombination event occurred in the genome of the single serotype IV strain clustering within CC6-8-10 that likely acquired the *cps* IV operon from ST-459 within an 85.4 kb genomic region.

We described independent and previously unreported large horizontal transfer events, involving the *cps* IV operon and resulting in the expansion of serotype IV through different CCs. While a capsular switch by homologous recombination of the *cps* locus is considered to be quite rare in GBS^42^, the exchange by conjugation of large genomic tracts that could include the *cps* operon has already been described to contribute to diversification in this species^18^. Overall, our results provide a detailed picture of the diversity and the evolution of the emerging serotype IV, underline the importance of a genomic surveillance of emerging clones and should be considered in designing containment strategies based on vaccination.

## Methods

### GBS isolates, growth conditions and genomic DNA preparation

The internal collection included 70 GBS type IV strains. Thirty-three isolates (n = 29 from vaginal-rectal swabs of colonizing pregnant women who delivered healthy babies and 4 from GBS-infected newborns) were collected during 2008–2010 in the eight European countries (Belgium, Bulgaria, Czech Republic, Denmark, Germany, Italy, Spain, and the UK) participating in the DEVANI consortium^43^. Four strains isolated from adults (two from carriers and two from patients with invasive disease, defined as isolation from a normally sterile site) were collected by the Istituto Superiore di Sanità (Rome, Italy) before 2006 and described in previous studies^44^. Three were from Portugal from adults with invasive disease (n = 2) and a carrier (n = 1)^40^. Thirty were from the US from adults or infants (n = 2) with invasive disease and colonized pregnant women (n = 4). Five of these strains were collected in Atlanta during 2000–2003, while the other 25 were collected in Houston, of which 11 were isolated during 2002–2006 and 14 from adults with bacteremia during 2012–2014 within a laboratory-based surveillance study of invasive GBS infection in adults (Edwards *et al*., submitted for publication).

GBS strains were grown at 37 °C in 5% CO_2_ in Todd Hewitt Broth (Difco Laboratories) or in trypticase soy agar supplemented with 5% sheep blood. Expression of the type IV capsular polysaccharide was confirmed for each strain by B-Strep-Latex test (Statens Serum Institut) following manufacturer’s instructions. Genomic DNA was isolated by a standard protocol for gram-positive bacteria, using the GenElute Bacterial Genomic DNA Kit (Sigma) according to the manufacturer’s instructions.

### Whole genome sequencing (WGS)

Whole genomic sequencing was performed on libraries generated using 1 ng of purified bacterial DNA processed by Nextera XT DNA Library preparation Kit (Illumina). Samples were normalized with a bead-based approach, and later pooled in an equal amount and finally diluted for the deep sequencing run following Nextera XT protocol. Sequencing was carried out on a HiSeq 2500 platform in a 100 bp paired-end run with the TruSeq SBS version 3 chemistry (Illumina).

### Genome assembly and annotation

A two-step approach was followed to assemble the 70 newly sequenced genomes. First a library of super-reads (36–54% of total reads) was obtained by merging paired-end reads of each genome with PEAR 0.9.5 (http://www.exelixis-lab.org/web/software/pear). Then each genome was assembled three times using SPAdes 3.5^45^ with a different sampling (1, 2 and 3 millions of paired reads) of all the sequenced reads as pair-end reads library and all the super-reads as single-end read library. The number of contigs, number of predicted genes, contig length and N50 were measured with QUAST 2.3^46^ and used to choose the best assembly. The resulting coverage ranged from 150x to 280x, with an average of 220x. All assemblies were annotated with Prokka^47^ using a genus specific non-redundant BLAST database, built from all the translated annotations retrieved from Ref Seq^48^ for TaxId 1311, clustered with CD-HIT^49^.

### Genome alignments, SNPs detection and phylogenetic analysis

The full genome dataset used for phylogenetic analyses included the 70 new type IV genomes, 415 draft and complete genomes retrieved both from the NCBI database and 6 from our internal collection. All the alignments, core genome and SNPs extractions were performed with Parsnp from the Harvest suite^50^, a rapid core genome multi-alignment software based on a suffix graph data structure for the rapid identification of maximal unique matches (MUMs). The software is a conservative core-genome aligner and SNPs extraction tool and was run with default parameters using a curated genome database.

Multiple alignments of the concatenated SNPs extracted from each core genome were used to build Maximum likelihood phylogenetic trees (1000 bootstrap replications), using the best scoring substitution model found by MEGA version 6.06^51^.

A global population tree was built based on 16,638 SNPs extracted from the core region of 491 genome sequences (533, 586 bp, 24.7% of the reference genome from strain 2603V/R), rooted on a piscine strain and plotted using the *ape* package^52^ in the R environment (http://www.r-project.org.).

An alignment of concatenated core sequences extracted from 130 CC17 and CC23 strains (1,500,718 bp, 72.8% of the reference genome NGBS572, ST-452) yielded 14,790 SNPs that were used to construct a neighbor-net split network using the software SplitsTree4^53^. Distances between taxa were estimated using the UncorrectedP method.

### Recombination analysis

SNPs density plots were built using harvest tools and visualized with Ginger (Harvest suite)^50^. The phylograms used in the plot were constructed from the alignment of the SNPs extracted from CC17 or CC23 strains and a ST-19 (CC19) strain used as outgroup to root the tree. Visual inspection of the high SNPs density areas on the chromosome map was used to define large recombination events. To confirm visual analysis results, average SNPs density per 100 bp was calculated for each of the identified regions using custom scripts in R. For statistical validation, a Bayesian analysis was performed using the software BratNextGen (http://www.helsinki.fi/bsg/software/BRAT-NextGen/)^54^. Briefly, a whole genome alignment of representative strains from CC17 (COH1, ST-17), CC23 (515, ST-23) and ST-452 (NGBS572) or CC1 (NGBS061, ST-459), CC-6-8-10 (A909 ST-7 and 5877, ST-10) was performed with progressive Mauve^55^, with the standard settings. A core alignment was built by filtering and concatenating Locally Collinear Blocks of size ≥1000 bp using the stripSubsetLCB script (http://darlinglab.org/mauve/snapshots/2015/2015-01-09/linux-x64/). Recombination analysis was performed with BratNextGen conducting 10 iterations and 100 permutations, fixing the hyperparameter to 1 with 0.05 as a P-value cut-off. The extracted SNPs and the identified recombination regions were then plotted on a complete circular reference genome using the software BRIG^56^.

### Sequence analysis

To confirm *cps*-type and assign PI variants to each of the 83 genomes an *in silico* typing was performed by BLAST analysis, using type IV *cps* operon (accession number: AF355776) and the pili backbone protein variants reference sequences (PI-1, locus tag SAG0645; PI-2a, SAG1407, locus tag PI-2b, SAN_1519) as query.

STs were assigned by comparing the genomic sequences with the 7 housekeeping genes of the GBS MLST system (http://pubmlst.org/sagalactiae/) using the Bio-MLST-Check-2.0.1510612 suite (http://search.cpan.org/~ajpage/Bio-MLST-Check-2.0.1510612/). The assignment to CCs was done as previously reported^23^. Variants of major virulence factors present in each strain were identified by BLASTn search using Geneious R8.1.6 (http://www.geneious.com). The nucleotide sequences used as query were retrieved from the NCBI database. The variant-specific sequences of Alp were retrieved from a published molecular typing scheme^57^. Gbs2018 variants were grouped into three major clusters according to a reported classification^58^. The distribution of the antibiotic resistance genes present in the Antibiotic Resistance Database^59^ and the RESFAMS database^60^ within the genomes of the 70 newly sequenced strains was performed using custom scripts based on the BLAST and HMMer algorithms (Supplementary Table S4).

## Additional Information

**Accession codes**: The newly sequenced genomes were deposited in the NCBI database under the BioProject ID PRJNA311314.

**How to cite this article**: Campisi, E. *et al*. Serotype IV *Streptococcus agalactiae* ST-452 has arisen from large genomic recombination events between CC23 and the hypervirulent CC17 lineages. *Sci. Rep.* **6**, 29799; doi: 10.1038/srep29799 (2016).

## Supplementary Material

Supplementary Information

## Figures and Tables

**Table 1:** *In silico* typing of 83 GBS type IV strains.

CC	ST	PI	Disease				no. of strains
			Adult	Neonatal	Colonization	n.a.	
1	2	1 + 2a	0	1	4	2	7
	3	1 + 2a	0	0	2	0	2
	136	1 + 2a	0	0	1	0	1
	196	1 + 2a	1	1	15	1	18
	288	1 + 2a	0	0	0	1	1
	459	1 + 2a	10	1	4	2	17
	499	1 + 2a	1	0	0	0	1
	533	1 + 2a	0	0	1	0	1
	589	1 + 2a	0	0	0	1	1
	645	1 + 2a	0	0	1	0	1
	new (s.l.v. ST-1)	1 + 2a	0	0	1	0	1
	new (s.l.v. ST-196)	1 + 2a	0	1	2	0	3
	n.a. (s.l.v. ST-2)	1 + 2a	0	0	0	1	1
6-8-10	10	1 + 2a	1	0	0	0	1
17	291	1 + 2b	1	0	0	0	1
	new (s.l.v. ST-291)	1 + 2b	1	0	1	0	2
	n.a. (s.l.v. ST-291)	1 + 2b	0	0	0	1	1
23	452	2b	12	2	4	2	20
	24	2b	1	0	0	0	1
	468	2b	1	0	0	0	1
	new (s.l.v. ST-452)	2b	1	0	0	0	1

Abbreviations: CC, Clonal Complex; ST, Sequence Type; PI. Pilus Island; n.a, not available; slv, single locus variant.
