# Supplementary material for: Serotype IV Streptococcus agalactiae ST-452 has arisen from large genomic recombination events between CC23 and the hypervirulent CC17 lineages
Source: Sci Rep. 2016 Jul 14;6:29799. doi: 10.1038/srep29799 (PMC4944191; doi:10.1038/srep29799)
Supplement: Supplementary Information [file srep29799-s1.pdf]

Serotype IV *Streptococcus agalactiae* ST-452 has arisen from large genomic recombination events between CC-23 and the hypervirulent CC-17 lineages

Edmondo Campisi[1,2], C. Daniela Rinaudo[1], Claudio Donati[3], Mara Barucco[1,4], Giulia Torricelli[1], Morven S. Edwards[5], Carol J. Baker[5,6], Imma Margarit[1], Roberto Rosini[1]*

[1]GSK Vaccines s.r.l., Siena, Italy.

[2]Sapienza, Università di Roma, Rome, Italy.

[3]Department of Computational Biology, Research and Innovation Centre, Fondazione Edmund Mach, San Michele all'Adige, Italy.

[4]Department of physics "Enrico Fermi", University of Pisa, Pisa, Italy.

[5]Department of Pediatrics, Baylor College of Medicine, Houston, Texas.

[6]Department Molecular Virology and Microbiology, Baylor College of Medicine, Houston, Texas.

[*] Address correspondence to roberto.x.rosini@gsk.com

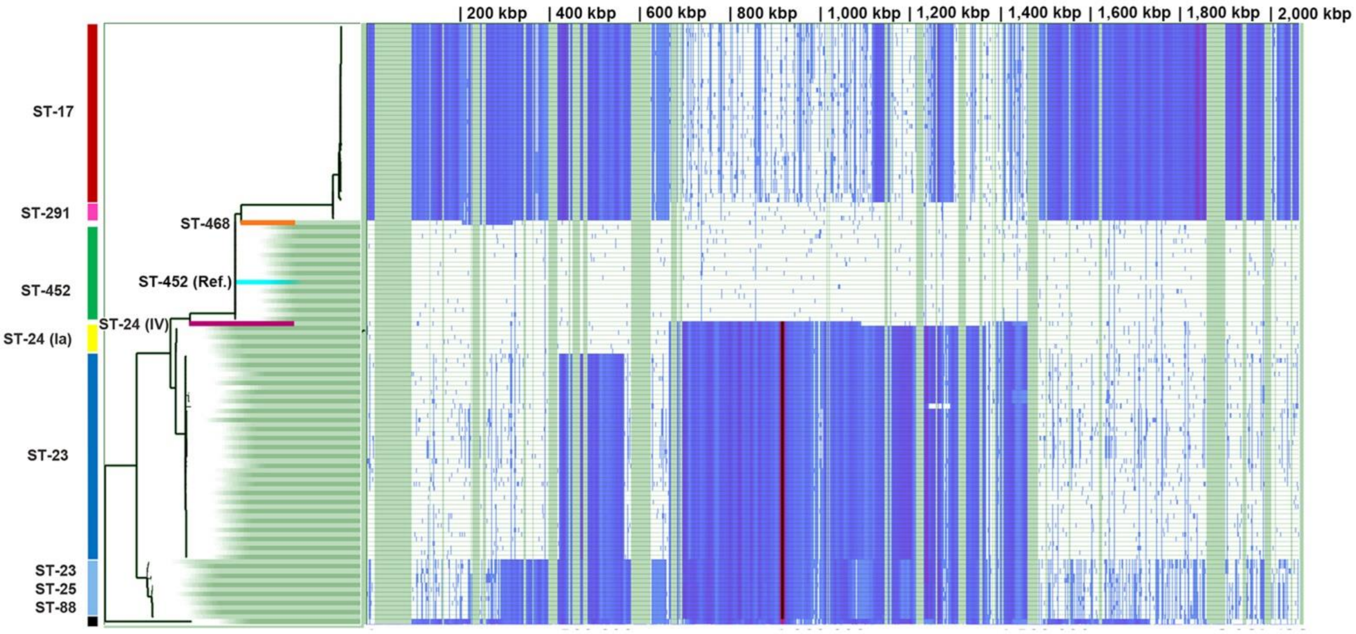

Figure S1. SNP density plot of CC17 and CC23 strains. Multiple-alignment view of the variant sites detected in the core genome of 130 GBS strains plotted on the complete chromosome of the ST-452 reference strain NGBS572 (type IV). The reconstructed phylogenetic tree (rooted on a ST-19 strain) is paired with its corresponding rows in the multi-alignment based on SNPs identified on the 72% of the core genome. Variations with respect to the reference ST-452 strain (indicated as “Ref.” within the tree) are represented by a density plot that reveals the phylogenetic signatures of the resulting clades. Highly conserved stretches of sequence appear as blank regions that suggest common evolution, while a high density of SNPs (purple-blue regions) indicates distantly related regions. Green areas represent non-core regions excluded from the analysis. Taxa are highlighted on the left side of the tree by vertical bars, colored according to their ST.

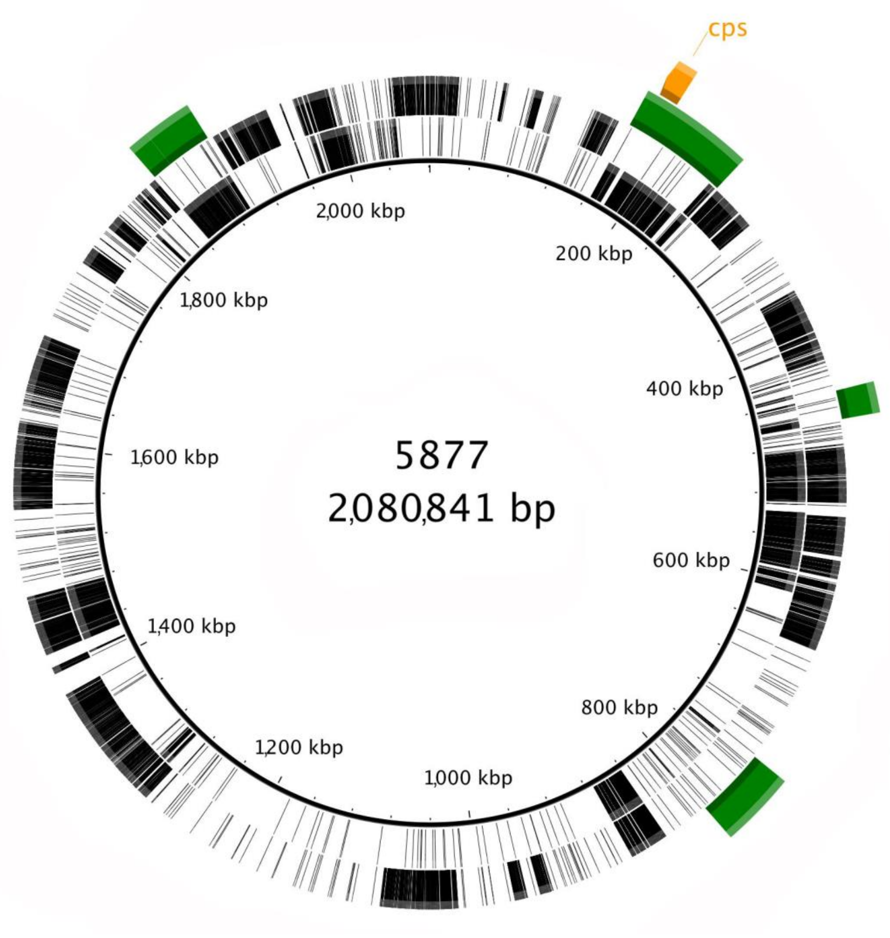

Figure S2. Identification of recombination areas in serotype IV ST-10 5877 strain. A909 (ST-7, CC-6-8-10) and NGBS061, CC1, ST459) genomes were compared to 5877 (ST-10, CC-6-8-10) for polymorphism detection. Polymorphisms identified in ST-7 strain A909 (innermost ring) and ST-459 strain NGBS061 (second ring) against the serotype IV ST-10 strain 5877 are depicted in black. Foreign genomic segments identified in the 5877 draft genome by Bayesian analysis of recombination (BRATNextGen) coming from serotype IV ST-459 strain NGBS061 are represented in green. Higher polymorphisms density at definite regions of the genome correlated with recombination identified between the two strains. An 85.4 Kb recombination region contains the *cps* operon (orange).

Table S1. Clinical and molecular data of the 70 sequenced GBS serotype IV strains.

Isolate name	ST	CC	Pili genotype	Host disease	Isolation year	Isolation source
1999	new	1	1 + 2a	Colonization	NA	NA
2274	3	1	1 + 2a	Colonization	NA	NA
5675	196	1	1 + 2a	Adult invasive disease	NA	NA
351521	2	1	1 + 2a	Colonization	2001	NA
AB-11	459	1	1 + 2a	Adult invasive disease	2012	Blood
AB-17	459	1	1 + 2a	Adult invasive disease	2012	Blood
AB-22	459	1	1 + 2a	Adult invasive disease	2012	Blood
AB-42	459	1	1 + 2a	Adult invasive disease	2013	Blood
AB-46	459	1	1 + 2a	Adult invasive disease	2013	Blood
AB-48	459	1	1 + 2a	Adult invasive disease	2013	Blood
AB-55	459	1	1 + 2a	Adult invasive disease	2013	Blood
AB-70	459	1	1 + 2a	Adult invasive disease	2014	Blood
BE-PW-052	196	1	1 + 2a	Colonization	2009	Vaginal-rectal
BE-PW-058	196	1	1 + 2a	Colonization	2009	Vaginal-rectal
BE-PW-073	196	1	1 + 2a	Colonization	2010	Vaginal-rectal
BE-PW-092	196	1	1 + 2a	Colonization	2010	Vaginal-rectal
BE-PW-118	196	1	1 + 2a	Colonization	2010	Vaginal-rectal
BE-PW-149	196	1	1 + 2a	Colonization	2010	Vaginal-rectal
BE-PW-150	196	1	1 + 2a	Colonization	2010	Vaginal-rectal
BE-PW-162	new	1	1 + 2a	Colonization	2010	Vaginal-rectal
BE-PW-163	196	1	1 + 2a	Colonization	2010	Vaginal-rectal
ATL-58	459	1	1 + 2a	Colonization	2002	Vaginal-rectal
CZ-NI-016	459	1	1 + 2a	Neonatal invasive disease	2010	Blood
CZ-PW-017	196	1	1 + 2a	Colonization	2009	Vaginal-rectal
CZ-PW-140	459	1	1 + 2a	Colonization	2009	Vaginal-rectal
CZ-PW-150	459	1	1 + 2a	Colonization	2010	Vaginal-rectal
DE-PW-196	2	1	1 + 2a	Colonization	2009	Vaginal-rectal
DK-NI-019	196	1	1 + 2a	Neonatal invasive disease	2010	Placenta fetal side
DK-PW-096	196	1	1 + 2a	Colonization	2010	Vaginal-rectal
DK-PW-161	2	1	1 + 2a	Colonization	2009	Vaginal-rectal
DK-PW-167	196	1	1 + 2a	Colonization	2010	Vaginal-rectal
ES-NI-012	2	1	1 + 2a	Neonatal invasive disease	2008	CSF
ES-NI-013	new	1	1 + 2a	Neonatal invasive disease	2008	Blood
ES-PW-028	645	1	1 + 2a	Colonization	2008	Vaginal-rectal
ES-PW-060	196	1	1 + 2a	Colonization	2008	Vaginal-rectal
ES-PW-083	new	1	1 + 2a	Colonization	2008	Vaginal-rectal

ES-PW-101	3	1	1 + 2a	Colonization	2008	Vaginal-rectal
ES-PW-130	196	1	1 + 2a	Colonization	2008	Vaginal-rectal
ES-PW-135	196	1	1 + 2a	Colonization	2008	Vaginal-rectal
ES-PW-185	196	1	1 + 2a	Colonization	2008	Vaginal-rectal
GB-PW-024	2	1	1 + 2a	Colonization	2009	Vaginal-rectal
IT-PW-075	459	1	1 + 2a	Colonization	2009	Vaginal-rectal
IT-PW-086	533	1	1 + 2a	Colonization	2009	Vaginal-rectal
IT-PW-097	136	1	1 + 2a	Colonization	2009	Vaginal-rectal
NOVUI-11	459	1	1 + 2a	Adult invasive disease	2005	Joint fluid
NOVUI-2	459	1	1 + 2a	Adult invasive disease	2005	Blood
SH4090	499	1	1 + 2b	Adult invasive disease	2008	NA
404562	new	17	1 + 2b	Adult invasive disease	2001	NA
BG-PW-065	new	17	1 + 2b	Colonization	2010	Vaginal-rectal
AB-13	468	23	2b	Adult invasive disease	2012	Blood
AB-24	452	23	2b	Adult invasive disease	2012	Blood
AB-35	452	23	2b	Adult invasive disease	2013	Blood
AB-47	24	23	2b	Adult invasive disease	2013	Blood
AB-56	452	23	2b	Adult invasive disease	2013	Blood
AB-7	452	23	2b	Adult invasive disease	2012	Blood
ATL-29	452	23	2b	Colonization	2003	Vaginal-rectal
ATL-77	452	23	2b	Colonization	2000	Vaginal-rectal
ATL-91	452	23	2b	Neonatal invasive disease	2003	NA
ATL-94	452	23	2b	Colonization	2003	Vaginal-rectal
IT-PW-052	452	23	2b	Colonization	2008	Vaginal-rectal
NOVUI-1	452	23	2b	Adult invasive disease	2005	Blood
NOVUI-10	new	23	2b	Adult invasive disease	2006	Blood
NOVUI-19	452	23	2b	Adult invasive disease	2002	Blood
NOVUI-26	452	23	2b	Neonatal invasive disease	2006	Blood
NOVUI-4	452	23	2b	Adult invasive disease	2004	Blood
NOVUI-5	452	23	2b	Adult invasive disease	2005	Bone
NOVUI-6	452	23	2b	Adult invasive disease	2005	Blood
NOVUI-7	452	23	2b	Adult invasive disease	2004	Blood
NOVUI-8	452	23	2b	Adult invasive disease	2004	Blood
5877	10	6-8-10	1 + 2a	Adult invasive disease	NA	NA

Abbreviations: ST = Sequence Type; CC = Clonal Complex; NA = Not Available; CSF = Cerebrospinal Fluid.

Table S2. List and accession numbers of the publicly available GBS genotype IV strains genomes included in the study.

Isolate name	ST	CC	Accession Number
BSU92	196	1	NZ_ALRJ00000000
CCUG 28551	unknown	1	NZ_ANDA00000000
GB00082	2	1	NZ_ALSR00000000
GB00901	459	1	NZ_ALUK00000000
GB00911	452	23	NZ_ALUN00000000
GB00933	452	23	NZ_ALUU00000000
Gottschalk 1005B	288	1	NZ_ALSJ00000000
LMG 15091	unknown	17	NZ_ALQX00000000
MRI Z1-012	2	1	NZ_ALRX00000000
MRI Z1-215	589	1	NZ_ANET00000000
NGBS061	459	1	CP007631
NGBS367	291	17	SRX507468*
NGBS572	452	23	CP007632

*Strain NGBS367 was assembled in house from SRA data.

Table S3. MLST allelic profiles of the new STs submitted to the *S. agalactiae* MLST database.

Isolate name	CC	*adhP*	*pheS*	*atr*	*glnA*	*sdhA*	*glcK*	*tkt*
1999	1	94	1	3	1	1	2	2
404562	17	2	new	1	2	1	1	1
BE-PW-162	1	1	1	new	1	1	12	2
BG-PW-065	17	2	25	new	2	1	1	1
ES-NI-013	1	new	1	3	1	1	12	2
ES-PW-083	1	new	1	3	1	1	12	2
LMG_15091	17	unknown	25	1	2	1	1	1
NOVUI-10	23	5	25	new	3	2	3	3

Abbreviations: CC = Clonal Complex; unknown = sequence of the gene not available;

Table S4. Antibiotic resistance genes within the 70 sequenced GBS serotype IV strains.

Isolate name	ST	Antibiotic resistance genes		
		**Aminoglycosides**	**Macrolides**	**Tetracyclin**
1999	new	none	none	*tetM*
2274	3	none	none	none
5675	196	none	none	*tetM*
351521	2	none	none	*tetM*
AB-11	459	none	*ermA*	*tetM*
AB-17	459	none	*ermA*	*tetM*
AB-22	459	none	*ermA*	*tetM*
AB-42	459	none	*ermA*	*tetM*
AB-46	459	none	*ermA*	*tetM*
AB-48	459	none	*ermA*	*tetM*
AB-55	459	none	*ermA*	*tetM*
AB-70	459	none	*ermA*	*tetM*
BE-PW-052	196	none	none	*tetM*, *tetO*
BE-PW-058	196	none	none	*tetM*
BE-PW-073	196	*aphIIIA*, *ant6*	*ermA*	*tetM*
BE-PW-092	196	none	none	none
BE-PW-118	196	*aphIIIA*, *ant6*	*ermA*	*tetM*, *tetO*
BE-PW-149	196	none	none	none
BE-PW-150	196	*aphIIIA*, *ant6*	*ermA*	*tetM*, *tetO*
BE-PW-162	new	*aphIIIA*, *ant6*	*ermA*	*tetM*
BE-PW-163	196	*aphIIIA*, *ant6*	*ermA*	*tetM*
ATL-58	459	none	*ermA*	*tetM*
CZ-NI-016	459	none	*ermA*	*tetM*
CZ-PW-017	196	*aadK*	none	none
CZ-PW-140	459	*aadK*	*ermA*	*tetM*
CZ-PW-150	459	*aadK*	*ermA*	*tetM*
DE-PW-196	2	none	none	none
DK-NI-019	196	*aadK*	none	*tetM*
DK-PW-096	196	*aadK*	none	none
DK-PW-161	2	none	none	*tetM*
DK-PW-167	196	*aadK*	none	*tetM*
ES-NI-012	2	*aadK*	none	*tetM*
ES-NI-013	new	*aadK*	none	*tetM*
ES-PW-028	645	none	none	*tetM*
ES-PW-060	196	*aadK*	none	*tetM*
ES-PW-083	new	*aadK*	none	*tetM*

ES-PW-101	3	*aphIIIA*, *ant6*, *aadK*	*ermA*	*tetM*
ES-PW-130	196	*aadK*	none	*tetM*
ES-PW-135	196	*aadK*	none	*tetM*
ES-PW-185	196	*aadK*	none	*tetM*
GB-PW-024	2	none	none	*tetM*
IT-PW-075	459	*aadK*	*ermA*	*tetM*
IT-PW-086	533	none	none	*tetM*
IT-PW-097	136	*aadK*	none	*tetM*, *tetO*
NOVUI-11	459	*aadK*	*ermA*	*tetM*
NOVUI-2	459	*aadK*	*ermA*	*tetM*
SH4090	499	*aadK*	none	*tetM*
404562	new	none	none	*tetM*
BG-PW-065	new	none	none	*tetM*
AB-13	468	none	none	*tetM*
AB-24	452	none	none	none
AB-35	452	none	none	none
AB-47	24	none	*mefA*	*tetM*
AB-56	452	none	none	none
AB-7	452	none	none	none
ATL-29	452	none	none	none
ATL-77	452	none	none	none
ATL-91	452	none	none	none
ATL-94	452	none	none	none
IT-PW-052	452	none	none	none
NOVUI-1	452	none	none	none
NOVUI-10	new	none	*mefA*	none
NOVUI-19	452	none	none	none
NOVUI-26	452	none	none	none
NOVUI-4	452	none	none	none
NOVUI-5	452	none	none	none
NOVUI-6	452	none	none	none
NOVUI-7	452	none	none	none
NOVUI-8	452	none	none	none
5877	10	*aadK*	none	*tetM*
